# Electricity, Neurology, and Noninvasive Brain Stimulation: Looking Back, Looking Ahead

**DOI:** 10.1155/2020/5260820

**Published:** 2020-04-13

**Authors:** Vijay Renga

**Affiliations:** Department of Neurology, Dartmouth Hitchcock Medical Center, Geisel School of Medicine at Dartmouth, One Medical Center Drive, Lebanon, NH 03756, USA

## Abstract

Electricity and neurology evolved synchronously over the past few centuries. This article looks at their origins and their journey into noninvasive brain stimulation technique of transcranial direct current stimulation (tDCS), which is now popular in neuroscience research.

## 1. Introduction

On a long journey, it is useful to look back and figure out how far we have come, to know which way we are heading. Centuries ago, electricity and neurology started a journey together which has taken us a long way. This article is a brief account of that journey into noninvasive brain stimulation.

From prehistoric times, electricity fascinated humans, whether it came as thunderous lightning bolts “from the heavens” or as electric sparks from rubbing stones. From time immemorial, we have attempted to harness the power of electricity. An electric fish applied to the scalp for treating headache was one of the earliest documented therapies. From violet ray to tDCS (transcranial direct current stimulation), there have been waves of interest in noninvasive brain stimulation. Attempts have been plentiful, results neither convinces nor ignorable. Some have enjoyed great success, while others have perished.

## 2. Part 1: Looking Back

### 2.1. Electricity: Origin

The word “Electricity” came from the Latin word “electricus” meaning “amber like,” amber being a Greek word. Materials like fur when rubbed against amber could accumulate enough static charge and create a spark of “electricity.” Thales of Miletus (600 BC), also known as the “Father of Science,” was the first person to study static electricity. This was in an era where anything that moved was alive, even if it were a lodestone attracted to iron by magnetism. William Gilbert (1544–1603), a physician to Queen Elizabeth I, who wrote volumes on magnetism coined the term “Electricity” [1].

### 2.2. The Electric Eel (*Electrophorus electricus*)

If we look further back, it is conceivable that the greatest contributor to electricity and neurology is the electric eel [2]. The characteristic feature of this fish is its ability to deliver an electric shock, either to its prey or to its opponent of such high magnitude as to kill them or numb them in an instant. It was called the “Thunderer of the Nile” for it could even immobilize a horse. There are several species of electric fish, with the torpedo fish and electric eel being the common ones. The numbing and shocking effects were used to treat headaches, seizures, and pain [3]. The fish made its way into ancient Greek, Roman, and Arabic medicine. The drawings of the fish can be found in Egyptian tombs [4].

### 2.3. Electricity: From Fish to Jar

Early researchers attempted to conduct the electricity generated by the fish into circuits using wires. Interest later evolved from conducting electricity to the generation of electricity. Otto von Guericke developed the first static generator in 1672 [5]. It got perfect over the next century, with the Leyden jar in 1742 becoming the most promising device. The first case of electric healing came soon after with Jean Jallabert, Professor of Physics in Geneva, who connected the paralyzed foot of a stroke victim to a Leyden jar and made his paralyzed extremity move. The patient improved weeks later and could use his extremity. In 1752, Benjamin Franklin attempted to treat epileptic fits using electricity from Leyden jar, and the therapy came to be known as “Franklinism” [6, 7].

### 2.4. Animal Electricity

The focal point setting forth the synchronous evolution of electricity and neurology was a serendipitous experiment in 1780 by Luigi Galvani, the famous Italian physicist and physician. Galvani was dissecting a dead frog on a table used for electrical experiments. The frog's leg kicked as his assistant touched the sciatic nerve with the scalpel. Unaware of the static currents picked up by the scalpel, Galvani thought an “animal electricity” had “manifested.” The finding created the notion of “bioelectricity” driving the muscle contraction, which got termed “Galvanism.” Giovanni Aldini, nephew and disciple of Galvani, was a strong proponent of “Galvanism.” He held public demonstrations of “Galvanism” where he stimulated the face, diaphragm, and extremities of a guillotined criminal to generate facial expression, breathing, and dancing movements [5, 8, 9]. The horror of watching the “dead come alive with electricity” became widespread. Mary Shelley's “Frankenstein” is a product of an era of “Franklinism” and “Galvanism” [10].

### 2.5. The Electric Era

The concept of “Galvanism” got refuted and proven wrong by Alessandro Volta. Based on the anatomic descriptions of the electric fish's nerve plates, he used dissimilar metal plates soaked in brine to invent the first electric “battery” [5, 11].

Faraday's experiment helped create alternating currents. We can find scores of reports during the following two centuries where electricity flourished in the treatment of neurologic and psychiatric conditions ranging from the use of Faradic currents to treat tabes by Joseph Babinski and “melancholy” treatment using voltaic battery by Aldini.

Nicola Tesla, a genius way ahead of his times, developed the alternating current from its origins by Michael Faraday. He met with Paul Oudin and conceived ideas of developing electrotherapeutic instruments. Paul Oudin built the “Violet Ray,” which found a variety of therapeutic uses from 1890 to 1910s [7]. An array of electrical devices ranging from violet rays for transcutaneous stimulation to Garceau nerve stimulator for direct cortical stimulation were made. However, these devices were proven nonbeneficial, got discontinued, or wiped out by great depression. Some of them also went into disrepute as “Quack Devices” ending up as exhibits like the ones at the Museum of Questionable Medical Devices at Bakken in Minneapolis.

Postdepression era saw a resurgence in electrostimulation techniques in psychiatry. An inverse relation was seen between schizophrenia and epilepsy. Treatment of schizophrenia involved inducing convulsions by injecting camphor or insulin, which were suboptimal, unsafe, and life-threatening. Ugo Cerletti and Lucio Bini watched butchers using electric pincers to cause convulsions and immobilize pigs in slaughterhouses [12, 13]. They adapted the technology to the field of psychiatry for electroconvulsive therapy (ECT), and it became the greatest electrical therapy to date.

### 2.6. Brain Polarization

Masked by the success of ECT, brain polarization experiments with smaller currents remained less prominent during this period. In the 1950s, there was renewed interest in peripheral nerve stimulation. Peripheral nerve polarization was shown to change the potentials recorded from the brain [14]. In addition, direct brain polarization studies in animals showed long-lasting changes in cortical excitability [15]. Influenced by these findings, polarization studies were conducted in humans that found significant benefits in depressed and psychotic patients resistant to other forms of treatment, including ECT [16–18]. Currents varying from 50 to 500 *μ*A were used, which showed that anodal currents improved alertness, mood, and motor activity, whereas cathodal polarization produced quietness and apathy. However, the interest in noninvasive stimulation faded after inability to replicate the results [17].

Cranial electrostimulation (CES) device which is a derivative of the earlier studies was used for the treatment of depression, fibromyalgia, and sleep till recently, when it got downgraded to a class III device by the FDA because of inadequate efficacy.

## 3. Part 2: Looking Ahead

### 3.1. Revival of Brain Polarization Using Noninvasive Measures

In a 1962 article in *Nature*, Bindman et al. described short polarizing currents on the cerebral cortex of the rat, which created prolonged changes in cortical excitability [15]. Decades later, Priori et al. studied these effects in humans using weak anodal and cathodal currents of <0.5 mA for 7 seconds with excitability changes determined by transcranial magnetic stimulation (TMS). Anodal followed by cathodal currents resulted in a lowered cortical excitability [19]. Nitsche et al. further refined this technique with stronger currents of 0.2–1.0 mA for a longer duration of 1–5 minutes and showed an increase in motor cortical excitability following anodal stimulation and a decrease in excitability with cathodal stimulation [20, 21]. The residual effects were detectable up to 90 minutes and sensorimotor and cognitive effects up to 30 minutes after stimulation. Long-term potentiation or depression is thought to be responsible for the long-lasting effects [22]. These findings paved the way for a flurry of new research into the field of transcranial direct current stimulation.

### 3.2. Polarizing the Brain Using tDCS

The materials required for tDCS are a constant current stimulator and scalp electrodes. These stimulators use currents of 1–4 mA. Electrodes are placed over predetermined sites based on the 10-20 EEG system [23, 24]. Direction of flow determines the polarization of the underlying tissues. When current goes from anodal pole to the reference electrode, it creates a negative field in the underlying brain tissue that lowers the threshold for depolarization. When the current is reversed, as in cathodal stimulation, an opposite effect of hyperpolarization and inhibition occurs. tDCS modulates the threshold for firing, without generating a neural impulse. Studies on brain models with current density distributions have shown that despite a large percentage of charge being shunted through the scalp, tDCS carries adequate currents to the underlying cortex to impact neuronal excitability [25]. Surface electrodes induced currents of around 1.5 mA are decipherable by intracranial electrodes during presurgical evaluation studies [26]. tDCS has been shown to change the regional blood flow of targeted sites and their connected regions [27].

### 3.3. Neuroplastic Effects of Brain Polarization

tDCS holds the potential for helping regeneration of injured brain cells and aids neuroplasticity [28–30]. It may also help regulate the interhemispheric inhibition from the contralesional hemisphere into the ipsilesional hemisphere. We can try to understand this concept with an exercise. Raise your right arm and try drawing the number “6” in the air while trying to draw a “9” with the other arm. The thing that is troubling you is the interhemispheric inhibition. In a healthy individual, there is a synchronous inhibition and facilitation that helps in bimanual tasks. That gains importance when there is an interhemispheric imbalance as in a stroke. The inhibitory contralesional hemisphere worsens ipsilesional hemispheric function. We have shown bi-hemispheric stimulation with cathodal inhibition over the normal hemisphere and anodal facilitation of the lesional hemisphere to improve functional recovery in chronic stroke patients [31].

### 3.4. tDCS as a Research Tool

During the past decade, there has been an explosion in tDCS-based studies. Being a low cost set up, minimal to no side effects, ease of operation, and availability of commercial devices, tDCS has become popular in research settings. There are over a thousand active trials going on with tDCS. In addition, newer techniques like tACS-transcranial alternating current stimulation, tRNS-transcranial random noise stimulation, and tPCS-transcranial pulsed current stimulation are being marketed [32]. Expensive high definition arrays devices to target focal areas limiting the current spread are also available.

From cognitive research to clinical applications, there are not many areas left in neuroscience, which tDCS has not touched by now. In addition, commercial devices that work like tDCS devices are being made available as “neuromodulation devices” to treat migraine, depression, pain, fibromyalgia, and other conditions, even though there are no FDA approved indications to use tDCS as a treatment modality.

### 3.5. tDCS as a Cognitive Enhancer

tDCS enhances a variety of cognitive tasks and performance, which includes working memory, decision making, motor learning, and speed and vocabulary, just to name a few. This led to the trials of tDCS in autistic disorders, mood disorders, dementia, and neuropsychiatric illnesses, which have all shown benefits [33]. tDCS devices adopted and commercialized for gaming head bands and cognitive enhancers are now available to purchase for the public. tDCS is also being used in military training for improving reaction times.

### 3.6. tDCS: Caveats

Even though tDCS has found widespread usage, the validity is being questioned [34]. The fundamental issue has been a lack of explanation on how the current passes through the skin and skull to the targeted brain tissue and creates only desired effects. The role of the magnetic fields created by tDCS on an electrical brain is still unclear [35].

## 4. Conclusion

We have a never-ending fascination with electricity and its application to neuroscience. Every few decades, there seems to be a rise and fall in a different version of the same technology. From the violet rays in the 1910s [7] to the polarization studies in the 1950s [16] and cranial electrotherapy stimulation (CES) devices in the 1980s, there has been much interest in the application of electricity to neurology. tDCS could be the new iteration for this decade, trying to unlock a potential that can achieve success akin to ECT. It remains to be seen if that promise delivers. For all we know—in this long journey—a small possibility remains that we are going around in circles.
